# Whole-patient conversations: Implementation of a practical positive psychiatry intervention for medical trainees

**DOI:** 10.1192/j.eurpsy.2021.1238

**Published:** 2021-08-13

**Authors:** J. Lam, M. Feingold-Link, J. Noguchi

**Affiliations:** 1 Student, Warren Alpert Medical School, Providence, United States of America; 2 Hospice/palliative Care, Warren Alpert Medical School, Providence, United States of America; 3 Director For Service-learning And Community Mentoring, Warren Alpert Medical School, Providence, United States of America

**Keywords:** Medical Education, resilience, Meaning in life, Positive Psychiatry

## Abstract

**Introduction:**

Positive psychiatry is the science and practice of psychiatry seeking to promote overall well-being and understand the “positive” aspects of the patient’s life, such as resilience, social connections, and meaning and values in life. While positive psychiatry research has recently blossomed, the field lacks practical ways to integrate these overarching principles into clinical practice. Life review interventions are commonly used in palliative care, spiritual care, and geriatrics, and involve a healthcare team member interviewing a patient about their life.

**Objectives:**

Our objective is to describe the implementation of a positive psychiatry-informed life story review initiative into medical education, with the goal of creating a structure for medical trainees to see the larger context of patients’ health, understand how past experiences influence current values, and improve patients’ overall well-being.

**Methods:**

First- and third-year students at Alpert Medical School of Brown University are required to participate in at least one strengths-based life review with a patient in the community or inpatient setting, transcribe the story, and integrate the story into the electronic health record.

**Results:**

Preliminary results demonstrate high acceptability and perceived development of patient-centered competencies, such as understanding patients as more complete human beings. While this is a low cost and sustainable intervention, barriers include buy-in from medical educators, hospital administrators, and trainees.

**Conclusions:**

To our knowledge, this is one of the first positive psychiatry-informed interventions to be implemented into the required medical curricula. Life story reviews may allow providers to understand the “positive” aspects of patients’ lives and understand their patients better as people.

